# Peripheral thyroid hormone levels and hepatic thyroid hormone deiodinase gene expression in dairy heifers on the day of ovulation and during the early peri-implantation period

**DOI:** 10.1186/s13028-016-0231-6

**Published:** 2016-09-08

**Authors:** Marie Margarete Meyerholz, Kirsten Mense, Matthias Linden, Mariam Raliou, Olivier Sandra, Hans-Joachim Schuberth, Martina Hoedemaker, Marion Schmicke

**Affiliations:** 1 Endocrinology Laboratory, Clinic for Cattle, University of Veterinary Medicine Hannover, Bischofsholer Damm 15, 30173 Hannover, Germany; 2 Institute for Farm Animal Reproduction, IFN Schönow e.V., Bernauer Allee 10, 16321 Bernau, Germany; 3 Faculty of Mathematics and Physics, Leibniz University, Welfengarten 1, 30167 Hannover, Germany; 4 Biologie du Développement et Reproduction, INRA, UMR1198, Domaine de Vilvert, 78350 Jouy-en-Josas, France; 5 Immunology Unit, University of Veterinary Medicine, Bischofsholer Damm 15, 30173 Hannover, Germany; 6 Clinic for Cattle, University of Veterinary Medicine, Bischofsholer Damm 15, 30173 Hannover, Germany

**Keywords:** Dairy heifer, Thyroid function, Early pregnancy, TSH, Triiodothyronine, DIO1 gene

## Abstract

**Background:**

Before the onset of fetal thyroid hormone production, the transplacental delivery of maternal thyroid hormones is necessary for embryonic and fetal development. Therefore, the adaptation of maternal thyroid hormone metabolism may be important for pregnancy success and embryo survival. The aims of this study were to determine the thyroid hormone levels during the early peri-implantation period until day 18 and on the day of ovulation, to determine whether pregnancy success is dependent on a “normothyroid status” and to determine whether physiological adaptations in maternal thyroid hormone metabolism occur, which may be necessary to provide sufficient amounts of biologically active T_3_ to support early pregnancy. Therefore, blood samples obtained on the day of ovulation (day 0) and days 14 and 18 of the Holstein–Friesian heifers (n = 10) during the respective pregnant, non-pregnant and negative control cycles were analyzed for thyroid-stimulating-hormone (TSH), thyroxine (T_4_) and triiodothyronine (T_3_). Liver biopsies (day 18) from pregnant and respective non-pregnant heifers were analyzed for mRNA expression of the most abundant hepatic thyroid hormone deiodinase (*DIO1*) by real time qPCR.

**Results:**

Although liver *DIO1* mRNA expression did not differ between the pregnant and non-pregnant heifers on day 18, the serum concentrations of TSH and T_3_ on day 18 were higher in non-pregnant heifers compared to pregnant heifers (P < 0.05). Moreover, T_3_ decreased between day 0 and 18 in pregnant heifers (P < 0.001).

**Conclusions:**

In conclusion, no associations between thyroid hormone patterns on day 18 and pregnancy success were detected. During the early peri-implantation period, TSH and T3 may be affected by the pregnancy status because both TSH and T_3_ were lower on day 18 in pregnant heifers compared to non-pregnant dairy heifers. In further studies, the thyroid hormone axis should be evaluated throughout the entire gestation to confirm these data and identify other possible effects of pregnancy on the thyroid hormone axis in cattle.

## Background

Thyroid hormones play a key role in fertility, conceptus development, tissue differentiation and fetal growth [1–5]. In humans and rats, the conceptus starts to produce thyroid hormones during the 2nd trimester of pregnancy [1, 5]. Afterwards, the maternal production of thyroid hormones is less essential for fetal development. However, before the onset of fetal thyroid hormone production, the transplacental delivery of maternal thyroid hormones is necessary [6, 7]. The importance of thyroid hormones for the bovine embryo was substantiated by results derived from in vitro studies that show that supplementation with T_3_ and T_4_ was associated with an improved viability of bovine embryos [8]. In addition, the hormonal activity of the thyroid gland has an important role in the metabolism of cells, lipids and carbohydrates as well as in the lactation course [9]. According to these data, on the one hand, pregnancy success might be dependent on a “normothyroid status,” and on the other hand, physiological adaptations in maternal thyroid hormone metabolism during early pregnancy in cattle might be necessary to provide sufficient amounts of biologically active T_3_ to support early pregnancy, including the growth and viability of the conceptus.

The thyroid hormone axis consists of the hypothalamic thyrotropin releasing hormone (TRH) and thyroid stimulating hormone (TSH), which are released by the pituitary. Under the control of TSH, the thyroid gland mainly produces the inactive pro-hormone T_4_, which can be activated locally through tissue-specific deiodinases (*DIO*) and deiodinated into the active hormone T_3_ [10]. By modulating *DIO* expression levels in different organs, e.g., the liver, adequate T_3_ concentrations can be achieved even during iodine deficiency [11]. In the liver, *DIO1* is expressed in high amounts [11], and its expression level influences the T_3_ blood concentration. Furthermore, the hepatic expression level of *DIO1* is known to be altered by steroid hormones, such as androgens in rats [12], and was previously shown to have higher expressed during the late pregnancy of pluriparous Holstein–Friesian cows than immediately postpartum [13]. Whether sexual steroid hormone patterns or early pregnancy may have an influence on hepatic *DIO1* expression has not been examined in cattle until now. McCann and Reimers [14] reported that cyclic heifers had higher serum concentrations of T_3_ and T_4_ during estrus compared to diestrus, whereas Elecko et al. [15] showed that the blood concentrations of T_4_ decreased under estradiol benzoate infusion. However, the effect of early pregnancy on the maternal thyroid hormone axis and hepatic *DIO1* expression has not been previously examined in heifers. Therefore, the aim of this study was to examine whether the maternal thyroid hormone axis differs between early pregnant and respective non-pregnant cycles of healthy Holstein–Friesian heifers.

## Methods

The experimental setup was previously published by Meyerholz et al. [16]. All of the interventional procedures were performed according to the German legislation on animal welfare and approved by the Lower Saxony Federal State Office for Consumer Protection 279 and Food Safety under the reference number AZ 33.14-42502-04-12/0744. Thirty Holstein–Friesian heifers aged 14.1 ± 1.6 months with a mean body weight of 337.8 ± 23.7 kg were used. The heifers were clinically healthy, and the health status was monitored throughout the entire experimental period. Briefly, three cycles of each of the Holstein–Friesian heifers (n = 10) were evaluated. Each cycle started on the day of ovulation (day 0) and ended on day 18. During the first “negative control” (NC) cycle, no embryo was transferred, whereas throughout the following cycles, the embryo transfer was performed on d 6 ± 12 h. Pregnancy was confirmed by high progesterone concentrations on day 18 and recovery of a trophoblast tissue by uterine flushing. Conversely, non-pregnant heifers showed estrus symptoms on day 18.

### Blood samples and liver biopsies

Blood samples were obtained from the jugular vein on days 0, 14 and 18, and due to the circadian and ultradian rhythmicity of thyroid hormones, the samples were always collected in the morning between 7:00 and 9:00 a.m. The 10 mL serum samples were collected in EDTA tubes (Sarstedt, Nümbrecht, Germany), maintained at room temperature (RT) for approximately 2 h and centrifuged for 10 min (1500×*g*, RT). After centrifugation, the serum and plasma was stored at −20 °C until further analysis. Liver biopsy specimens (10 mm × 3 mm) were collected on day 18 in sterile Eppendorf cups, which were immediately frozen in liquid nitrogen and stored at −80 °C until mRNA extraction. The relative abundance of hepatic *DIO1* in comparison with the housekeeping genes *GAPDH* and *RPS9* (Table 1) was measured by real-time PCR as previously described [17].

**Table 1:** The quantitative real-time PCR primers for DIO1 and two housekeeping genes in the liver biopsy specimens of pregnant and non-pregnant heifers on day 18

Gene symbol	Primer	Primer sequence (5′→3′)	Accession number	Start	Product bp
*GAPDH*	Forward	caa cat caa gtg ggg tga tg	NM_001034034.1	315	202
	Reverse	ggc att gct gac aat ctt ga		516	
*RPS9*	Forward	gat tac atc ctg ggc ctg aa	NM_001101152	340	201
	Reverse	cag gga gaa gtc gat gtg ct		540	
*DIO1*	Forward	ccg tgg tgg tag aca caa tg	NM_001122593.1	605	204
	Reverse	tca ggt tgg gca cct aga ac		808	

*GAPDH* glyceraldehyde-3-phosphate dehydrogenase; *RPS9* ribosomal protein S9; *DIO1* deiodinase-1

### Blood parameters

#### TSH [ng/ml]

For the ELISA, an antibody targeted against bovine TSH (anti-bovine TSH, 1:10 pre-diluted, AFP-642482Rb) was obtained from the National Hormone and Peptide Program (NHPP, National Hormone and Peptide Program, NIDDK and Dr. Parlow) and was diluted and used at a final dilution of 1:2500. The standard curve ranged from 0.2 to 100 ng/ml bovine TSH (AFP-8755B, obtained from the NHPP, NIDDK and Dr. Parlow) and was dissolved in peptide buffer. The standards, controls and plasma samples (in triplicate) were added to a microtiter plate coated with the antibody, and the plate was then incubated for 24 hat RT. After washing the plate, biotin-labeled TSH [biotin labeled TSH (AFP-8755B, obtained from the NHPP, NIDDK and Dr. Parlow)] was added to all of the wells and incubated for 3 h. Then, a streptavidin–horseradish peroxidase solution (Sigma Aldrich, St. Louis, MO, USA) was added, the substrate (containing tetramethylbenzidine, Sigma Aldrich, St. Louis, MO, USA) was pipetted after washing, and the reaction was stopped after 15 min by adding sulfuric acid (2M; Sigma Aldrich, St. Louis, MO, USA). The optical density was obtained at a wavelength of 450 nm, and the concentrations were calculated using Magellan software with the cubic spline modus (Magellan 3.11, Dortmund, Germany). The intra-assay CV was determined by measuring one bovine sample 20 times, and the result was 15.4. The lowest detection limit of the ELISA was 0.6 ng/ml, which was determined by using the last detectable concentration of a bovine serum sample that was serially diluted.

#### Thyroxine and triiodothyronine [nmol/l]

The total serum T_4_ and T_3_ concentrations were determined using radioimmunoassays (TOTAL T4 RIA KIT, IM1447-IM3286 and TOTAL T3 RIA KIT, IM1699-IM3287, Immunotech, Beckman Coulter, CA, USA). The assays were performed according to the manufacturer instructions, and the samples were analyzed in duplicate. The intra-assay CV was determined by analyzing the bovine serum samples within one test run; the CV was 6.2 % for T_4_ and 6.3 % for T_3_, and the inter-assay CV was calculated by analyzing the same bovine serum sample in ten different test runs. The inter-assay CV was 8.6 % for T_4_ and 7.7 % for T_3_. The lowest detection limits were 13.0 nmol/l for T_4_ and 0.3 nmol/l for T_3_.

### Statistical analyses

The Statistical Analysis System software (9.3, SAS Inc., Cary, NC, USA) was used to perform all of the statistical analyses. A linear mixed-effect model for repeated measures was chosen to work out the dependence of measured bovine thyroid hormone concentrations (T4, T3 and TSH) on varying groups (p, np, NC) and time (days 0, 14, 18). To meet the primary assumption of the restricted maximum likelihood method (REML), data was tested for normal distribution (PROC UNIVARIATE NORMAL) for each of the nine levels of group x time interaction. As data was predominantly normally distributed, PROC MIXED and method REML with fixed effects “group”, ‘time” and “group × time” and linear regression effect “age” were performed.

**Fig. 1:**
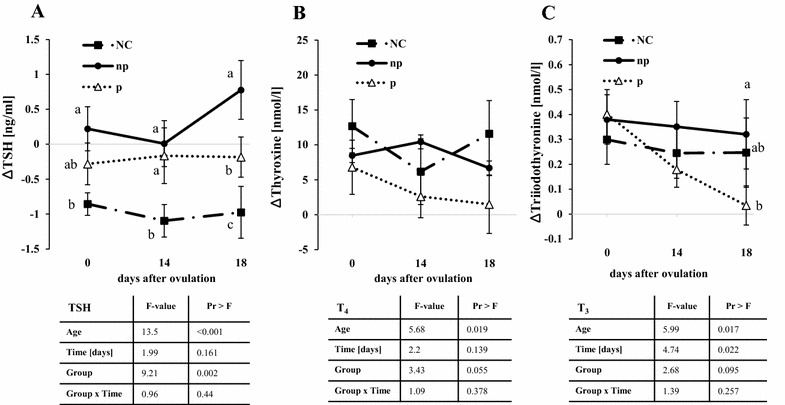
Age adjusted **A** thyroid-stimulating-hormone (∆TSH), **B** thyroxine (∆T_4_) and **C** triiodothyronine (∆T_3_) blood concentrations (mean ± SEM) of pregnant (p) heifers and respective non-pregnant (np) and negative control cycles (NC); the significant differences between the groups on certain days are marked with *different letters*. The results of the mixed–model are shown in the table below each graph

The age effect components in the linear mixed-effect model were estimated with the following intercepts and slopes: TSHest(age) = 8.1496–0.01016*age (P < 0.001), T4est(age) =−2.1811 + 0.08157*age (P = 0.019), T3est(age) = 0.7432 + 0.001949*age P = 0.017). The results throughout the manuscript are presented as mean ± standard error of the mean (SEM). Concerning T4, T3 and TSH concentrations, data was adjusted according to the linear regression component of the model (age). Therefore, differences were formed: “hormone concentration at each data point” minus “estimated concentration at age at sample day” resulting in “∆ hormone concentrations” (Fig. 1). Hepatic mRNA expression was compared using a paired Student’s *t* test (PROC TTEST PAIRED) and results are presented as mean ± SEM as well. For all procedures, the statistical significance was pre-established at P < 0.05. P values between P > 0.05 and P < 0.10 were considered statistical tendencies. F and P values for the fixed effects are presented below each graph (Fig. 1), significant differences between pregnant (p) negative control (NC), and non-pregnant (np) cycles are indicated by different letters (Fig. 1) whereas differences along the time points within each group are described in the text.

## Results

### Blood parameters

The thyroxine concentrations were higher in non-pregnant compared to pregnant cycles (P = 0.046) on day 14 but comparable at all of the other tested days. Triiodothyronine concentrations decreased in pregnant heifers between days 0 and 14 (P = 0.027) as well as between days 0 and 18 (P = 0.008). Interestingly, in non-pregnant heifers, T3 was higher on d 18 compared to pregnant heifers (P = 0.013). TSH concentrations were higher in non-pregnant compared to pregnant heifers on day 18 (P = 0.008) and during the NC (P < 0.001) (Fig. 1A–C).

### Hepatic mRNA expression

The relative abundance of *DIO1* mRNA expression on day 18 was comparable between pregnant (ΔCT 33.32 ± 4.35) and non-pregnant heifers (ΔCT 27.02 ± 7.78).

## Discussion

The first aim of the present study was to detect the possible associations between pregnancy success and the thyroid status on the day of ovulation; however, no differences in TSH, T_3_ or T_4_ levels were detected on the day of ovulation. Second, the maternal thyroid hormone axis was evaluated during early pregnancy in dairy heifers. TSH concentrations were lower in pregnant heifers compared to non-pregnant heifers on day 18; however, the negative controls showed even lower TSH levels compared to pregnant heifers. Therefore, an effect of early pregnancy on TSH is unlikely. However, it should be noted that daily blood sampling may not be adequate to evaluate the TSH concentration in heifers as thyroid hormones have a circadian and ultradian rhythmicity in bovine plasma [18]. In future studies, the TSH concentration and release should be evaluated during the first month of pregnancy because the chosen time period in this study could have been too short to observe the effect of thyroid axis adaptions during pregnancy in cattle. Conversely, collecting more blood samples per day or 24 h measurements could help identify the possible effects of pregnancy on TSH release.

Moreover, due to the experimental setup, the original data could have reflected the growth and pregnancy effects at the same time, indicating that NC differs from np cycles because the NC cycle was the first cycle to be performed during the experiment and the heifers were younger, whereas during the np and p cycles, the animals were comparable in age, but the maintenance of early pregnancy led to differences (data not shown). To minimize the effect of age on the data as a confounding variable, the regression factor “age” was calculated, hormone concentrations are presented as adjusted values and the effect was also statistically considered.

T_4_ concentrations tended to be lower in p compared to np on day 14. These results were unexpected, as studies in humans show elevated T_4_ levels during pregnancy [19, 20], which were possibly due to the thyroid stimulating effects of human chorionic gonadotropin (hCG) [21]. In cattle, a comparable effect cannot be indicated by the results of the present study.

Hepatic *DIO1* activity did not differ between p and np, irrespective of the higher T_3_ serum concentrations in non-pregnant heifers on day 18. Cann and Raimers [14] detected lower T_4_ and T_3_ levels during the luteal phase in heifers compared to the follicular phase [14]. In the present study, the T_3_ concentration decreased from days 0 to 18, which indicated a corresponding result concerning the steroid hormone influence on T_3_; however, no differences between p and np were detected concerning T_4_ concentrations in the present study. It would be interesting to determine in future studies whether the decreasing T_3_ concentration is originating from enhanced inactivation of T_3_ into reverse T_3_. Those inactive forms of T_3_ were not detected by the T_3_ assay used in the present study, and a separate analysis would be of interest in future studies in cattle. Type 2 deiodinase (*DIO2*) [22] was shown to be more highly expressed in the placental tissues of rats and humans during early pregnancy and could result in an inactivation of T_3_ to reverse-T_3_ or 3.3′-diiodothyronine [22, 23]; therefore, *DIO2* could represent a possible explanation for the lower T_3_ concentration in pregnant heifers compared to non-pregnant heifers. Placental *DIO2* measurement was not addressed in the present study but should be determined in future studies. In rats, pregnancy results in a decrease of total T_4_ and T_3_ concentrations throughout gestation [24], which might be in accordance with the present study; however, the thyroid hormone concentrations should be followed for a longer time period of pregnancy to substantiate the present preliminary data.

Another possible explanation for the lower T_3_ concentrations during early pregnancy could be an enhanced renal iodine clearance resulting from increased blood flow to the kidney and elevated glomerular filtration as observed in humans [25]. However, data concerning pregnancy-dependent elevated urinary excretion of iodine remain contradictory [26–28] and could be addressed in heifers to clarify the causes for T_3_ changes detected in the present study.

## Conclusions

In conclusion, TSH and T_3_ were lower on day 18 in pregnant compared to non-pregnant dairy heifers and indicate an effect of pregnancy on the thyroid gland axis. However, the underlying cause of the lower T_3_ concentrations remains unclear as at least the expression of hepatic DIO1, the most abundant deiodinase also responsible for differences in differenced in blood T_3_, was comparable. Based on our data, it seems interesting to focus on thyroid hormone metabolism in high producing dairy cows.
